# Management of Odontogenic Infections in Pregnant Patients: Case-Based Approach and Literature Review

**DOI:** 10.3390/pathogens14101024

**Published:** 2025-10-09

**Authors:** Andrei Hramyka, Agata Wieczorkiewicz, Jakub Bargiel, Krzysztof Śliwiński, Krzysztof Gąsiorowski, Tomasz Marecik, Paweł Szczurowski, Grażyna Wyszyńska-Pawelec, Jan Zapała, Michał Gontarz

**Affiliations:** 1 Students’ Scientific Group, Department of Cranio-Maxillofacial Surgery, Jagiellonian University Medical College, 30-688 Cracow, Poland; andrei.hramyka@student.uj.edu.pl (A.H.); a.wieczorkiewicz@student.uj.edu.pl (A.W.); 2 Department of Cranio-Maxillofacial Surgery, Jagiellonian University Medical College, 30-688 Cracow, Poland; jakub.bargiel@uj.edu.pl (J.B.); krzysieksliwinski3@wp.pl (K.Ś.); krzysztof.gasiorowski@uj.edu.pl (K.G.); tomasz.marecik@uj.edu.pl (T.M.); pawel.szczurowski@uj.edu.pl (P.S.); grazyna.wyszynska-pawelec@uj.edu.pl (G.W.-P.); jan.zapala@uj.edu.pl (J.Z.)

**Keywords:** odontogenic abscess, pregnancy complications, antibiotic therapy, dental infections

## Abstract

Background: Odontogenic abscesses may significantly affect maternal health during pregnancy. Aim: This study analyzes three cases of pregnant patients with odontogenic infections, comparing them to a control group of non-pregnant women, and reviews recent literature. Materials and Methods: Between January 2020 and April 2025, 3 pregnant and 70 non-pregnant women with odontogenic abscesses were treated. Clinical presentation, pathogens, therapy, and outcomes were compared. Results: Severe sequelae, such as rapid abscess spread and systemic inflammation, were more frequent in pregnant women, though not statistically significant (*p* = 0.068). Pregnant patients also tended toward prolonged intubation (*p* = 0.194) and targeted antibiotic use (*p* = 0.133). Antibiotic selection was based on gestational age, with beta-lactams preferred. Surgical interventions were more extensive, often involving multiple neck spaces. Hospitalization was longer (≥4 days in most cases) due to maternal–fetal monitoring. Conclusions: Odontogenic abscesses in pregnancy require individualized management and gestation-adjusted antibiotic therapy.

## 1. Introduction

Neglecting dental issues can lead to abscesses and periapical inflammation, both of which are potential sources of systemic infection and may contribute to the onset of systemic inflammatory response syndrome (SIRS). In addition to immunocompromised individuals, pregnant women are a particularly vulnerable group who require special dental care. Physiological changes during pregnancy can increase the risk of severe complications, which may impact fetal development or even pose a risk to the life of the fetus [1].

Hormonal homeostasis is closely linked to systemic regulation, with gonadal steroids such as estrogen and progesterone modulating immune function via receptors on nearly all human immune cells [2]. Dysregulated immune response and elevated levels of inflammatory biomarkers promote bacterial growth and increase susceptibility to inflammatory conditions [3]. Although abscesses are relatively uncommon during pregnancy, the most frequently observed oral inflammatory disorders in pregnant women include dental caries, gingivitis, and periodontitis [4]. Without adequate treatment, these infections can lead to a range of severe complications, including cellulitis, Ludwig’s angina (LA), orbital or cerebral abscesses, airway obstruction, mediastinitis, necrotising fasciitis, and even sepsis [5]. Potential adverse outcomes include the need for preterm cesarean delivery, low birth weight, intensive care for either the mother or the newborn, and, in rare cases, fetal or maternal death. Additionally, fetal pain has been reported in isolated cases [6].

The aim of the present study was threefold: to describe three cases of pregnant patients with odontogenic face and neck inflammatory conditions (abscesses, LA, phlegmon), to compare their clinical management and outcomes with a control group of 70 non-pregnant women treated for odontogenic abscesses in our department, and to contextualize the findings through a review of the relevant literature.

## 2. Materials and Methods

**Figure 1:**
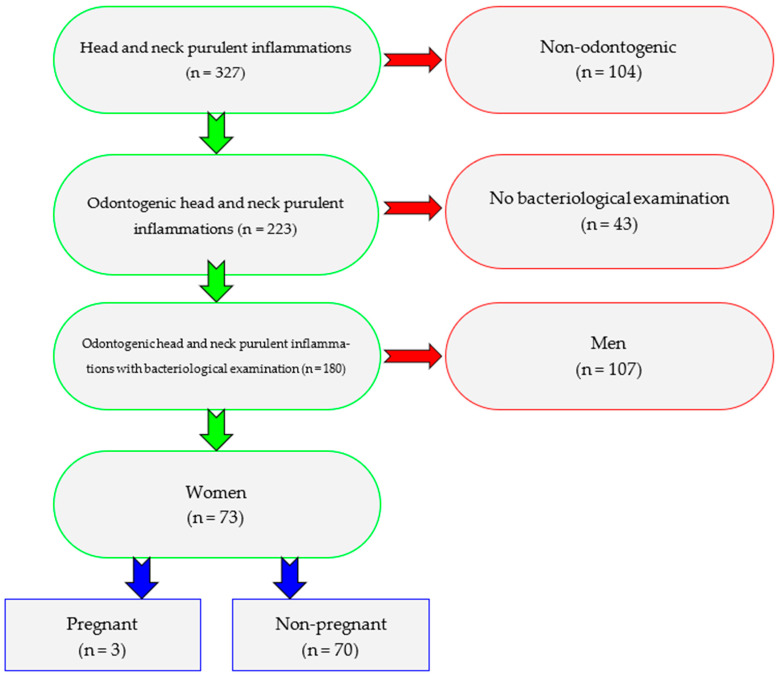
Flow chart of the study population selection.

In the period from January 2020 to April 2025, 327 adult patients (>18 y.o.) with head and neck inflammatory condition were treated in the Department of Cranio-Maxillofacial Surgery of the Jagiellonian University in Cracow, Poland. The inclusion criteria in the study were in series (Figure 1):

- Existence of odontogenic abscess.
- Presence of bacteriological examination.
- Female sex.
- Pregnancy.

In the final division there were 107 men and 73 women, including 3 pregnant women among them. Consequently, men were excluded from the comparative analysis, because the primary objective was to analyze clinical features and treatment outcomes of odontogenic abscesses specifically in pregnant patients, for whom pathophysiology, treatment considerations, and maternal–fetal safety differ from the general population. All patients in the study were of Caucasian ethnicity. 

This research was approved by the Institutional Review Board of Jagiellonian University (No 188.0043.1.503.2024). Patient consent was waived due to the retrospective nature of the study, as long as confidentiality of all personal data was provided. A comprehensive analysis of medical records was performed considering the following aspects: site of abscess and the involved teeth, clinical manifestations, bacteriological examination, antibiotics, surgical treatment, postoperative complications and outcome. Gynecological and obstetrical information for pregnant patients was mentioned, and clinical outcomes were compared between pregnant and non-pregnant women. A phlegmon has been defined as a diffuse lesion that has the capacity to propagate along connective tissue and muscle fiber. The distinction between an abscess and a phlegmon was based on the number of adjacent spaces involved in the inflammatory process: 1–2 bound spaces were classified as an abscess, whereas involvement of ≥3 spaces was defined as a phlegmon.

The distribution of quantitative variables was assessed for normality using the Shapiro–Wilk test. Chi-squared test (with Yates correction for 2 × 2 tables) or Fisher’s exact test (in case of low expected values) were used for comparisons of qualitative variables between groups. Student’s t-test was used for comparisons of quantitative variables between two groups. Significance level was set to 0.05. No formal corrections for multiple comparisons were applied, and results should therefore be interpreted with caution in the context of multiple testing. All the analyses were conducted in R software, version 4.5.1.

**Figure 2:**
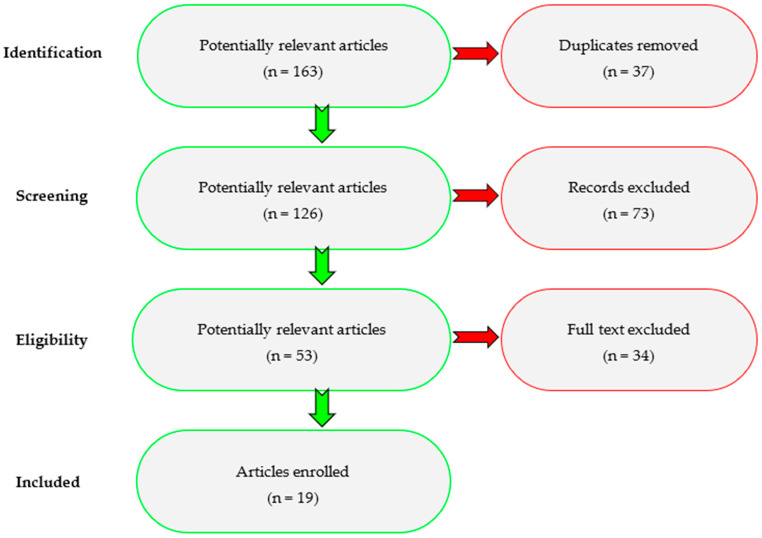
The flow diagram of a search procedure.

The literature review was conducted to identify cases of odontogenic infections in pregnant women. PRISMA principles were followed where applicable. However, narrative review with systematic search elements were implemented due to the scope of the study. Searches for articles were performed in the PubMed and Embase databases from January 2010 to April 2025. The following keywords and Boolean operators were used: (“odontogenic inflammation” OR “odontogenic abscess” OR “odontogenic infection” OR “dental inflammation” OR “dental abscess” OR “dental infection” OR “oral inflammation” OR “oral abscess” OR “oral infection”) (“pregnancy” OR “pregnant “ OR “gestation”) AND (“treatment” OR “management” OR “therapy” OR “surgery” OR “antibiotic”). The exclusion criteria in the study were in series (Figure 2):

- Duplicates.
- Non-odontogenic head and neck infections, studies unrelated to pregnancy.
- No full text, reviews, editorials, unclear results.

The literature screening and data extraction were performed independently by two reviewers (M.G. and J.B.). Any discrepancies were resolved through discussion, and, if necessary, adjudicated by a third reviewer (G.W.-P.).

A total of 163 articles were identified during the first search and 126 after the removal of duplicates. After title and abstract screening, 73 of these articles were excluded as not relevant. The full text of the remaining 53 papers was read and evaluated for eligibility, and 19 papers were included in the study for a total of 63 patients.

## 3. Results

### 3.1. Cases Presentations

#### 3.1.1. Case No. 1

**Figure 3:**
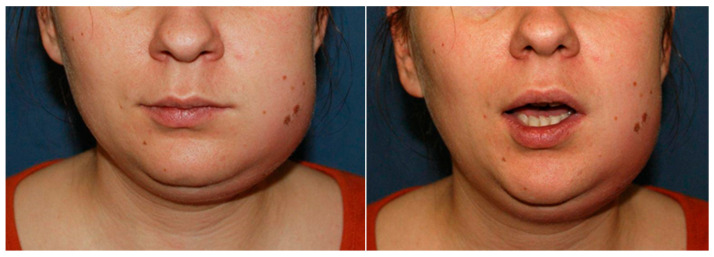
Clinical presentation of a 28-year-old pregnant woman with left-sided odontogenic submandibular and buccal abscesses.

A 28-year-old woman at 16 + 2 weeks gestation was admitted with left cheek and submandibular abscess, which developed 19 days after the extraction of teeth 28 and 38. Gravida 3 Para 1 Abortions 1 Living children 1 (G-P-A-L), indicating three pregnancies, one live child, one abortion, and one living child (G3 P1 A1 L1) were noted. The patient was afebrile at presentation but reported pain rated 4/10 on the Visual Analog Scale (VAS), trismus, and facial swelling. Her medical history was unremarkable (Figure 3). 

Gynecological consultation confirmed a single live fetus on both transabdominal (TA) and transvaginal (TV) ultrasound. There was no evidence of placenta previa or abruption. The patient underwent surgical intervention under general anesthesia for the management of a spreading odontogenic abscess. A microbiological swab was obtained for culture. The patient’s recovery was uneventful. Empiric antibiotic therapy included cefuroxime, and the patient was discharged in good general condition after 2 days of hospitalization. Bacteriological culture results were negative, and no further antibiotic treatment was required. The patient experienced transient trismus (grade II), which improved progressively as the wound healed. In total after 15 days of hospitalization the patient was discharged. Both the postoperative course and the pregnancy remained uneventful, with ongoing gynecological monitoring. 

#### 3.1.2. Case No. 2

A 34-year-old woman (G4 P2 A1 L2) at 35 weeks and 5 days of gestation was admitted to our department with a 12-day history of a facial phlegmon originating from a necrotic pulp of tooth 47. Previous outpatient treatment was unsuccessful due to insufficient drainage and deficient root canal therapy. The facial phlegmon extended into the pterygomandibular, masseteric, temporal and infratemporal spaces. 5/10 on the VAS, accompanied by trismus, swelling, and no fever. The patient had hypothyroidism. TA-USG and TV-USG revealed a single live fetus with no abnormalities. 

A multidisciplinary team performed surgery under general anesthesia because of advanced pregnancy and a critical local infection. Antibiotic therapy: clindamycin was changed to ceftriaxone. The patient was in our department for 8 days, in a stable condition, with no complications related to the pregnancy. The phlegmon spaces were irrigated daily with sodium bicarbonate, yet there was still temporal swelling with purulent discharge. Bacteriological results revealed the presence of Prevotella buccae, Prevotella nigrescens, Streptococcus anginosus and Streptococcus massiliensis. Clinical pharmacology recommended switching to piperacillin/tazobactam, as this was not contraindicated in the third trimester. 

**Figure 4:**
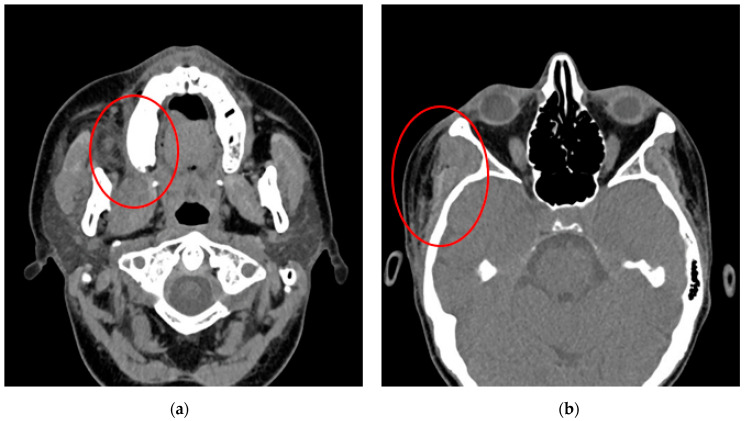
CT scans of a 34-year-old pregnant woman showing facial phlegmon involving the right pterygomandibular (**a**) and temporal spaces (**b**) (red circles).

On the seventh postoperative day, the patient was transferred to the Clinic of Obstetrics and Perinatology in good general condition. A cesarean section was performed with the patient’s consent due to the onset of labor, a previous cesarean section and cervical cerclage, recent abscess surgery and a suspected uterine scar defect. A healthy male child was delivered. No complications were encountered during the operation. Purulent drainage from the right submandibular area, swelling of the right temporal region and trismus were significantly greater on the 1st day after cesarean section. Breastfeeding was temporarily stopped due to possible dysbiosis. Control computed tomography revealed multiple abscesses in the right temporal and pterygomandibular spaces, as well as bone defects and periosteal reactions (Figure 4). A reoperation involving revision of the phlegmon spaces was performed on the second post-cesarean day. No complications occurred. In total after 15 days of hospitalization the patient was discharged with good general condition. 

#### 3.1.3. Case No. 3

**Figure 5:**
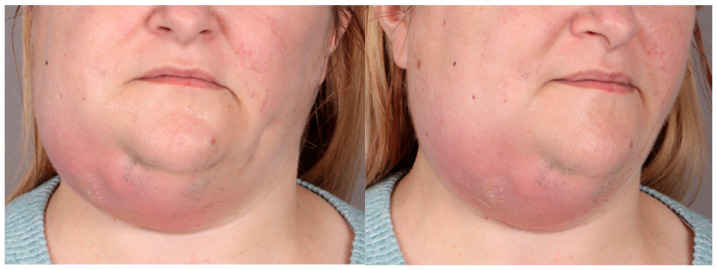
Clinical presentation of a 32-year-old pregnant woman with right-sided Ludwig’s angina involved the floor of the mouth.

A 32-year-old woman was referred to the department with a right-sided LA and no fever, having reported pain and swelling associated with teeth 46 and 47, which had necrotic pulp (Figure 5). The patient’s past medical history only noted high blood pressure; no medications or allergies were known. Despite the patient’s assertion that she was not pregnant, a gynecological evaluation revealed an 18 + 6-week gestation (G6 P5 A0 L5) that had not been detected. TA-USG revealed a single live fetus with no abnormalities.

In total, the patient underwent three operations due to difficulties in achieving complete drainage of the infected spaces. The 1st procedure was performed urgently to evacuate purulent discharge from the right perimandibular and submental regions. Unfortunately, just a few hours after extubation, the patient required oxygen therapy, resuscitation and emergency intervention due to respiratory distress and temporary cardiac arrest. The patient stabilized and was taken for the second surgery, which resulted in tracheostomy due to significant tongue base and laryngeal edema. The patient was subsequently transferred to the Intensive Care Unit (ICU), where a multidisciplinary approach was adopted. Samples for cultures were obtained, and treatment was adjusted according to an individual care plan. The empiric antibiotic—clindamycin, was started. Viable intrauterine gestation has been confirmed by USG.

On ICU day 3, she was successfully weaned from mechanical ventilation. Meanwhile, bacteriological testing revealed Moryella indoligenes and Streptococcus viridans group (except *S. pneumoniae*). Targeted antibiotics—initially ceftriaxone, subsequently ceftazidime, were prescribed. However, the patient required a 3rd revision surgery. The patient was gradually roused and, over a period of 3 days, was discharged from the ICU to our department. After that local improvement was noted and hospitalization time, in total, was 12 days, including 2 days of intubation. Later at 32 weeks of pregnancy, a cesarean section was performed on the patient, delivering a healthy female infant.

### 3.2. Pregnant vs. Non-Pregnant Women

Table 1 presents a comparison of patients treated for odontogenic abscesses in our department over the past 5 years. The analysis did not reveal any statistically significant differences between the groups. Due to a small sample size group, *p*-values should be interpreted with caution. In the pregnant group, there appeared to be a tendency toward a more aggressive course of inflammation, often involving multiple anatomical spaces (*p* = 0.068). This was associated with a greater need for prolonged intubation, ICU hospitalization, and targeted antibiotic therapy. Additionally, infections in pregnant patients were more frequently caused by multiple teeth with necrotic pulp. The type of bacteria isolated and the number of microorganisms cultured were comparable between pregnant and non-pregnant women. 

## 4. Discussion

Pregnancy often leads to oral health complications, which, if they are not treated properly, can have a negative effect on the pregnant person’s wellbeing. The presence of periodontal disease during pregnancy has been linked to preterm birth and low birth weight risks. Despite the proven safety and advantages of treatment, many pregnant women do not receive proper dental care [7,8,9]. The evidence indicates that non-surgical periodontal therapy is associated with a reduced risk of preterm birth, underscoring the importance of timely intervention [10,11]. The limited options for antibiotics and analgesics in pregnancy require healthcare providers to work together to achieve optimal management because of fetal safety concerns [12]. The risk factors for periodontitis in pregnant women include gestational age and parity, as well as poor oral hygiene practices. However, predictive tools exist to help identify patients who need preventive care [13]. The implementation of prenatal total oral rehabilitation programs has been shown to enhance maternal and infant health outcomes through improved oral health literacy and utilization of dental care [14]. Most obstetricians are aware of the importance of oral health, yet routine dental referrals remain limited due to insufficient training and systemic challenges. These challenges require integrated prenatal oral health screening and education [15,16]. The progression of infections is heavily dependent on immune dysregulation. Therefore, the use of targeted immunomodulatory therapies that utilize biomarkers has the potential to enhance the treatment outcomes of sepsis from odontogenic infections during pregnancy [17].

The rare odontogenic infection such as LA or phlegmon, which can occur during pregnancy, poses a severe threat to both maternal and fetal survival through airway obstruction and systemic complications [18,19]. The patient’s survival is dependent on prompt multidisciplinary intervention, which necessitates immediate surgical drainage and airway control measures [18,20]. Prolonging non-surgical treatment can have a negative impact on maternal and fetal health outcomes [21]. The management of advanced infections requires complex airway strategies combined with regional anesthesia methods to decrease potential risks [22,23]. Deep neck space infections require urgent surgical drainage in addition to intravenous antibiotics to ensure patient survival [24]. Continuous fetal monitoring through coordinated maternal-fetal care has been shown to reduce adverse outcomes [25].

Early pregnancy oral hygiene status together with periodontal disease acts as a major risk factor which leads to worsening oral health conditions during later pregnancy stages [26]. Untreated severe infections in the first and second trimesters cause systemic involvement that leads to submandibular cellulitis and intracranial abscesses and potentially results in fetal demise [27,28]. Surgical treatment together with antibiotic therapy at the right time helps prevent preterm labor and fetal distress during these gestational periods [29,30,31]. Preventive dental care together with early intervention becomes essential to stop the development of fatal infections [32].

During the third trimester, the risk of odontogenic abscess increases, which in turn is associated with a higher likelihood of preterm birth, fetal growth restriction, and premature rupture of membranes [26,33]. At this stage odontogenic infections may trigger acute fetal distress which requires immediate delivery (cesarean section) because they cause maternal respiratory failure according to reports of LA and cervicofacial cellulitis [30,34,35]. The primary treatment approach includes surgical drainage together with antibiotics (β-lactams, clindamycin, metronidazole) under close fetal monitoring to guarantee maternal and neonatal safety [35,36]. The combination of obesity and inadequate oral hygiene practices increases these risks which demonstrates the necessity of consistent dental care during pregnancy [26].

The study demonstrated a significant trend towards a more aggressive course of face and neck odontogenic inflammation in pregnant patients, frequently involving multiple anatomical spaces. This clinical pattern was associated with an increased incidence of prolonged intubation, admission to the ICU, and the requirement for targeted antibiotic therapy.

As outlined in previous studies, comparable observations have been reported regarding the severity of inflammatory processes during pregnancy. Our review of the literature revealed that odontogenic purulent inflammation occurred predominantly during the third trimester of pregnancy. Nearly half of the reported cases developed complications requiring ICU admission, with an average ICU stay of seven days and an average overall hospital stay of 15 days. Table 2 presents a comprehensive overview of the relevant literature on this problem.

The main limitation of the study is its retrospective design and the fact that it was conducted at a single clinical center, which restricts the generalizability of the findings. Furthermore, the very small number of pregnant patients (n = 3) substantially limited statistical power and increased the risk of a type II error, meaning that potentially important differences may not have reached statistical significance. The results may be influenced by random variation and heavily dependent on individual observations. The small sample size reflects how rare odontogenic inflammatory conditions are in pregnant patients. In most developed countries, potentially infectious dental issues are usually resolved before pregnancy in order to ensure optimal oral health. Despite this, the findings suggest a potential association between pregnancy and a more aggressive course of odontogenic infection. Given the limitation of only three cases, this study attempted to compare a group of non-pregnant patients with pregnant patients identified through a literature review, as presented in Table 2. However, many of the reviewed articles lacked sufficient clinical detail to allow for a meaningful comparison between the two groups. Additionally, the cases included in Table 2 involved patients of various ethnic backgrounds and primarily consisted of single case reports describing severe odontogenic infections with complications, which may introduce a significant risk of bias.

## 5. Conclusions

This study compared the course of odontogenic infections in pregnant and non-pregnant women, highlighting the potential risks posed to both mother and fetus. Although rare, odontogenic abscesses during pregnancy may progress aggressively and require a multidisciplinary treatment approach that balances maternal needs with fetal safety. Emphasis on early detection, preventive care, and awareness among both dental professionals and expectant mothers is crucial, with regular dental visits playing a key role in maintaining oral hygiene and reducing the risk of severe complications. The novelty of our work lies in the combined comparative analysis with a control group and the integration of a literature review.

## Figures and Tables

**Table 1:** Statistical analysis of characteristics in pregnant and non-pregnant women.

Parameter		Pregnant Women (N = 3)	Non-Pregnant Women (N = 70)	*p*-Value
Age [years]	Mean (SD)	31.33 (3.06)	42.6 (18.27)	*p* = 0.293
	Median (quartiles)	32 (30–33)	39 (27–55.75)	
	Range	28–34	18–86	
	n	3	70	
VAS	<5	1 (33.33%)	13 (18.57%)	*p* = 1
	≥5	2 (66.67%)	33 (47.14%)	
	Unknown	0 (0.00%)	24 (34.29%)	
Infected spaces	1	1 (33.33%)	60 (85.71%)	***p * = 0.068**
	≥2	2 (66.67%)	10 (14.29%)	
Bacteriological test	Positive	2 (66.67%)	55 (78.57%)	*p* = 0.53
	Negative	1 (33.33%)	15 (21.43%)	
Anaerobic bacteria	Present	2 (66.67%)	28 (40.00%)	*p* = 0.564
	Absent	1 (33.33%)	42 (60.00%)	
Aerobic bacteria	Present	2 (66.67%)	39 (55.71%)	*p* = 1
	Absent	1 (33.33%)	31 (44.29%)	
Bacterial species	0–1	1 (33.33%)	38 (54.29%)	*p* = 0.595
	≥2	2 (66.67%)	32 (45.71%)	
Number of teeth extracted	0–1	1 (33.33%)	54 (77.14%)	***p * = 0.148**
	≥2	2 (66.67%)	16 (22.86%)	
Empirical antibiotic therapy	Yes	3 (100.00%)	69 (98.57%)	*p* = 1
	No	0 (0.00%)	1 (1.43%)	
Targeted antibiotic therapy	Yes	2 (66.67%)	15 (21.43%)	***p * = 0.133**
	No	1 (33.33%)	55 (78.57%)	
Hospitalization time	<4 days	1 (33.33%)	44 (62.86%)	*p* = 0.554
	≥4 days	2 (66.67%)	26 (37.14%)	
Intubation	Yes	1 (33.33%)	4 (5.71%)	***p * = 0.194**
	No	2 (66.67%)	66 (94.29%)	

*p*—Qualitative variables: chi-squared or Fisher’s exact test. Quantitative variables: Student’s *t*-test.

**Table 2:** Pregnant women with odontogenic purulent inflammations: a literature review.

First Author/Year of Publication	No. of Cases	Age (Average)	Gestation Trimester	Type of Abscess	Bacteriological Examination	Hospitalization—Days (Average)	ICU Stay—Days	Complications
Moorhead/2010 [18]	1	24	3rd	LA	polymicrobial	9	8	ARDS, C-section
Hobson/2011 [28]	1	35	2nd	pterygomandibular	polymicrobial	14	14	subdural empyema, recurrent brain abscesses (frontal, temporal, and parietal lobe); Broca’s aphasia and apraxia with right hemiplegia
Wong/2012 [29]	5	22–33 (28.4)	1st (*n* = 2), 3rd (*n* = 3)	submandibular (*n* = 3), buccal (*n* = 1), sub masseteric and buccal (*n* = 1)	no data	1–6 (3)	1 (*n* = 1), 3 (*n* = 1)	C-section (*n* = 1)
Kamath/2012 [34]	1	24	3rd	LA	polymicrobial	21	no	C-section, reoperation
Celebi/2013 [30]	1	28	3rd	perimandibular and masticator space	no data	refused to be admitted	no	C-section
Wazir/2013 [31]	28	17–30 (24.8)	1st (*n* = 6), 2nd (*n* = 8), 3rd (*n* = 14)	LA (*n* = 10), submandibular (*n* = 8), submandibular and submental (*n* = 4), buccal (*n* = 2), masseteric (*n* = 2), submental (*n* = 1), maxillary canine (*n* = 1)	no data	no data	no data	no
Mukherjee/2013 [27]	1	38	3rd	phlegmon	no data	no data	no data	intrauterine fetal demise
Dalla Torre/2013 [24]	1	25	3rd	submandibular	polymicrobial	around 7 weeks	around 3 weeks	maternal ARDS and sepsis, intrauterine fetal demise, abscesses at the skull base, around the trachea and esophagus, mediastinitis and pleural empyema
Tocaciu/2017 [36]	1	29	2nd	phlegmon	no data	no data	3	no
Pereira/2017 [32]	1	30	3rd	buccal and submasseteric	monomicrobial	around 6 weeks	no	no
Ali/2019 [21]	10	18–35 (26.5)	2nd (*n* = 4), 3rd (*n* = 6)	submandibular (*n* = 7), LA (*n* = 2), submental (*n* = 1)	no data	1–22 (6.3)	no	necrotizing fasciitis (*n* = 2), delivery (*n* = 1)
Trahan/2020 [20]	1	34	3rd	LA	polymicrobial	9	5	C-section
Aziz/2020 [35]	3	31, 24, 28, (27.7)	3rd (*n* = 3)	submandibular (*n* = 2), submental (*n* = 1)	monomicrobial	13, 17, 8 (12.7)	no	preterm delivery (*n* = 1)
Shamim/2021 [22]	1	40	2nd	LA	no data	no data	no	intraoperatively failed intubation
Pucci/2021 [6]	1	36	3rd	submandibular	no data	around 2 weeks	no	C-section
Rahman/2022 [23]	1	25	3rd	LA	no data	4	no	no
Balaji/2024 [19]	1	26	3rd	LA	monomicrobial	12	6	C-section
Jain/2024 [25]	1	28	2nd	phlegmon	negative	5	no	no
Present study	3	28, 32, 34 (31)	2nd (*n* = 2), 3rd (*n* = 1)	phlegmon (*n* = 1), perimandibular (*n* = 1), LA (*n* = 1)	monomicrobial (*n* = 1), polymicrobial (*n* = 1)	2, 11, 15 (9.3)	2 (*n* = 1), no (*n* = 2)	C-section (*n* = 1), reoperation (*n* = 2)
**Total**	**63**	**18–40 (29.5)**	**1st (*n* = 8),** **2nd (*n* = 18),** **3rd (*n* = 37)**	**one space (*n* = 33),** **two spaces (*n* = 7),** **LA (*n* = 19),** **phlegmon (*n* = 4)**	**monomicrobial (*n* = 6),** **polymicrobial (*n* = 6),** **negative (*n* = 1),** **no data (*n* = 50)**	**1–49 (average 15; *n* = 31),** **no (*n* = 1),** **no data (*n* = 31)**	**1–21 (average 7; *n* = 9),** **no (*n* = 25),** **no data (*n* = 29)**	**C-section/delivery (*n* = 10),** **intrauterine fetal demise (*n* = 2)** **reoperations, ARDS or sepsis, brain abscesses or empyema, necrotizing fasciitis,** **no (*n* = 32)**

LA—Ludwig’s angina, ARDS—acute respiratory distress syndrome.

## Data Availability

The data were obtained from patients operated on the Cranio-Maxillo-Facial Surgery Department of Jagiellonian University, Cracow, Poland, and cannot be shared in accordance with the General Data Protection Regulation (EU) 2016/679.

## Abbreviations

The following abbreviations are used in this manuscript:

SIRS	Systemic Inflammatory Response Syndrome
G-P-A-L	Gravida–Para–Abortions–Living children
TA-USG	Transabdominal–Ultrasonography
TV-USG	Transvaginal–Ultrasonography
ICU	Intensive Care Unit
LA	Ludwig’s angina
ARDS	Acute Respiratory Distress Syndrome
